# Effect of a Novel Hydroxybenzoic Acid Based Mitochondria Directed Antioxidant Molecule on Bovine Sperm Function and Embryo Production

**DOI:** 10.3390/ani12070804

**Published:** 2022-03-22

**Authors:** João Campos Santos, Carla Cruz Marques, Maria Conceição Baptista, Jorge Pimenta, José Teixeira, Liliana Montezinho, Fernando Cagide, Fernanda Borges, Paulo J. Oliveira, Rosa M. L. N. Pereira

**Affiliations:** 1 Biotechnology and Genetic Resources Unit, INIAV—National Institute of Agrarian and Veterinarian Research, Quinta da Fonte Boa, 2005-048 Vale de Santarém, Portugal; joaopcsantos20@gmail.com (J.C.S.); carla.marques@iniav.pt (C.C.M.); conceicao.batista@iniav.pt (M.C.B.); jorge.pimenta@iniav.pt (J.P.); 2 CIVG, Center for Investigation Vasco da Gama (CIVG), Department of Veterinary Sciences, Escola Universitária Vasco da Gama, 3020-210 Coimbra, Portugal; liliana.montezinho@euvg.pt; 3 CIISA—Centre for Interdisciplinary Research in Animal Health, University of Lisboa, Av. da Universidade Técnica, 1300-477 Lisboa, Portugal; 4 CNC-Center for Neuroscience and Cell Biology, CIBB—Centre for Innovative Biomedicine and Biotechnology, IIIUC—Institute for Interdisciplinary Research, University of Coimbra, 3004-504 Coimbra, Portugal; jcsteixeira8@gmail.com (J.T.); pauloliv@cnc.uc.pt (P.J.O.); 5 MitoTAG, Biocant Park—Parque Tecnológico de Cantanhede, Núcleo 04, Lote 04, 3060-197 Cantanhede, Portugal; 6 CIQUP/Department of Chemistry and Biochemistry, Faculty of Sciences, University of Porto, Campo Alegre, 4169-007 Porto, Portugal; fernandocagide@yahoo.es (F.C.); fborges@fc.up.pt (F.B.)

**Keywords:** sperm, oocytes, mitochondria, mitochondria-targeted dietary antioxidants, ROS, ART

## Abstract

**Simple Summary:**

Gametes are particularly susceptible to oxidative stress that impairs the reproductive function. This study was conducted to study the effect of a mitochondria-targeted dietary antioxidant (AntiOxBEN2) on bovine sperm function. Different doses of this antioxidant were tested during spermatozoa capacitation and/or fertilization processes. Spermatozoa mitochondrial function was improved when AntiOxBEN2 was supplemented to the capacitation medium. Supplementation of both capacitation and fertilization media with AntiOxBEN2 (lowest dose) improved the fertilization process and embryo production. Our results showed that AntiOxBEN2 can be used for the prevention of oxidative stress in bovine spermatozoa and may constitute a putative novel therapeutic strategy to improve the outcomes of assisted reproductive techniques.

**Abstract:**

Sperm cells are particularly vulnerable to reactive oxygen species (ROS), impairing their fertilizing ability. Our objective was to study the effect of a novel mitochondrial-directed antioxidant, AntiOxBEN2, on bovine sperm function. This antioxidant was added to the semen capacitation medium (CAP), during the swim-up process, and to the fertilization medium (FERT) during the co-incubation of matured oocytes and capacitated spermatozoa, in concentrations of 0 (control), 1, and 10 µM. After the swim-up, sperm motility (CASA and visual analysis), vitality (eosin-nigrosin), mitochondrial membrane potential (JC1), intracellular ROS, adenosine triphosphate (ATP) levels, and basal metabolism (Seahorse Xfe96) were evaluated. Embryo development and quality were also assessed. Higher cleavage rates were obtained when 1 µM AntiOxBEN2 were added to CAP and FERT media (compared to control, *p* < 0.04). A positive effect of AntiOxBEN2 on intracellular ROS reduction (*p* = 0.01), on the increment of mitochondrial membrane potential (*p* ≤ 0.003) and, consequently, on the sperm quality was identified. However, the highest dose impaired progressive motility, ATP production, and the number of produced embryos. The results demonstrate a beneficial effect of AntiOxBEN2 (1 µM) on sperm capacitation and fertilization processes, thus improving embryonic development. This may constitute a putative novel therapeutic strategy to improve the outcomes of assisted reproductive techniques (ART).

## 1. Introduction

Male and female germ lines are particularly susceptible to oxidative stress that impairs the reproductive function. Classically, oxidative stress occurs when there is an imbalance between reactive oxygen species (ROS) production and the protective capacity of the cellular antioxidant machinery. In spermatozoa, several sources of ROS were previously identified [1]. Sperm mitochondria are highly prone to electron leakage during oxidative phosphorylation leading to ROS generation [1,2]. An unregulated elevation in ROS production causes damage to several essential molecules, such as lipids, polysaccharides, proteins, and DNA, leading to cell death by apoptosis/necrosis [3,4]. Depending on where the imbalance in ROS production/elimination occurs, oxidative stress can have several repercussions. Spermatozoa are particularly sensitive to the damaging effects of ROS since their cell membranes are composed of large amounts of unsaturated fatty acids that can be oxidized and contain small amounts of antioxidant enzymes that able to neutralize ROS, such as hydrogen peroxide (the glutathione peroxidase/reductase system or catalase) and O2^•−^ (superoxide dismutase). Therefore, high concentrations of ROS lead to the suppression of sperm function and to infertility problems [1,2,5,6].

In fact, sperm functioning requires the steady production of mitochondria-derived ATP. Therefore, sperm are rich in those organelles, which inevitably lead to increased ROS production [6]. Changes in spermatozoa’s DNA, plasma and mitochondrial membranes, and a decrease in their motility and vitality have been reported in the presence of high levels of ROS [6,7,8]. If low disequilibrium levels between ROS and cellular defenses exist, sperm cells can maintain their fertilization ability. However, when cell damage occurs, it can be transmitted to the next generation [7,9]. Particular attention should be given to assisted reproductive techniques (ART), so that the impact of ROS on the number and quality of produced embryos may be reduced, given the harmful effect they have on both oocytes and spermatozoa [10]. Several factors can increase oxidative stress during the in vitro process, including light, oxygen concentration, and the handling of oocytes, sperm, and embryos [11,12,13]. Therefore, an efficient solution to prevent and minimize oxidative stress should be attempted to improve ART outcomes.

In light of the major role that oxidative stress plays in the etiology of impaired sperm and oocyte function, it has been suggested that antioxidants may have a therapeutic role in the context of both in vivo and in vitro fertilization [1,10]. Moreover, the recognition of the essential role of mitochondria in the maintenance of cell life and of the mitochondrial dysfunction observed in several pathological states [14,15] encouraged the study of new mitochondria-targeted interventions. Despite this, the discovery and use of potential mitochondrial drugs is a challenge because they require a specific target affinity (drug delivery to mitochondria) and the guarantee that they do not present mitochondrial toxicity.

Hydroxybenzoic acids (HBA) are present in relatively high amounts in plants and fruits, and have several beneficial biological properties (antioxidant, anti-inflammatory, antimicrobial, anticancer, and neuroprotective) [16,17,18]. The antioxidant activity of HBAs has been associated with their chelating and sequestering properties of ROS, acting in the prevention of lipid peroxidation and in the inhibition of pro-oxidant enzymes involved in ROS production. The use of HBAs in human and veterinary therapy, alone or as adjuvants, is restricted due to limitations related to their bioavailability, pharmacokinetics, and selectivity, which are linked to their physicochemical properties (e.g., low lipophilicity) and fast metabolism. Several studies have been carried out to increase HBAs’ lipophilicity and stability and to improve their availability for intracellular targets. Recently, we developed a mitochondria-targeted antioxidant derived from HBA (AntiOxBEN2) by conjugation with tetraphenylphosphonium (TPP+) [4]. The antioxidant molecule, AntiOxBEN2, presented high lipophilicity, stability, and selectivity for the mitochondria with positive effects in different experimental tests performed using other cell models [4,19]. Moreover, preliminary results indicate that this antioxidant improves the rate of oocyte maturation and embryo cleavage, when supplemented with the maturation medium [20], making this molecule a putative novel therapeutic strategy.

A growing interest in controlling oxidative stress and its implication in numerous conditions, such as fertility, justified the need to evaluate new agents capable of modulating spermatozoa functioning. In this study, we investigated, for the first time, the effect of a novel mitochondrial-directed antioxidant, AntiOxBEN2 [4,19], in the prevention of oxidative stress of bovine spermatozoa during both the capacitation and the fertilization processes. Spermatozoa motility, vitality, ATP and ROS production, oxygen consumption, mitochondrial membrane potential, fertilization, and embryonic developmental ability were assessed. Herein, for the first time, our promising data demonstrate a beneficial effect of AntiOxBEN2 at 1 µM on both sperm capacitation and fertilization processes, and, as a result, improving embryonic development. Further studies should deepen the benefits of AntiOxBEN2 supplementation on sperm quality and metabolism.

## 2. Materials and Methods

### 2.1. Chemicals and Reagents

Cell culture medium, water for embryo culture, media components, chemicals, and reagents were purchased from Sigma-Aldrich (St. Louis, MO, USA) unless otherwise specified.

### 2.2. Synthesis of AntiOxBEN2

**Figure 1:**
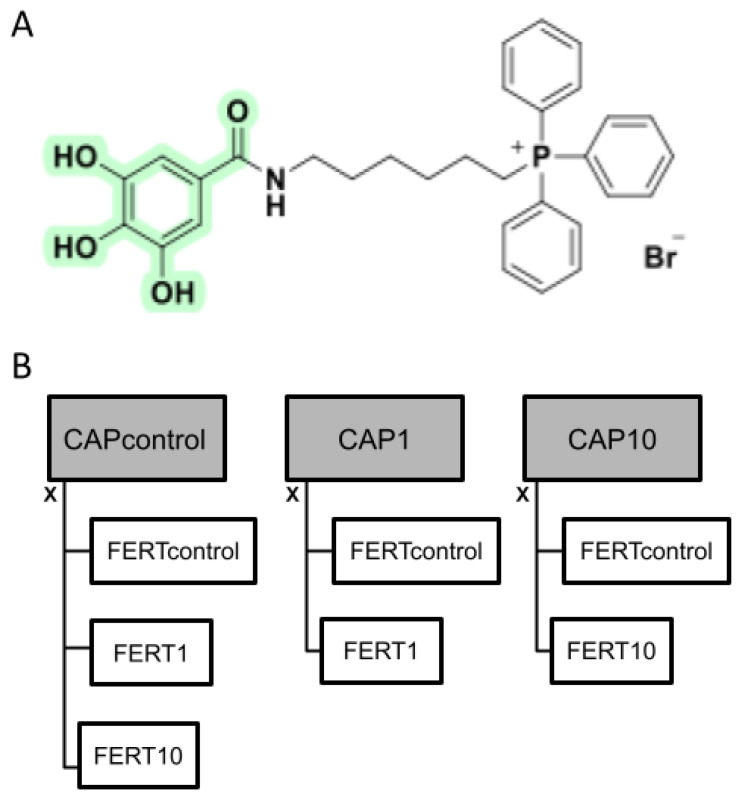
(**A**) Chemical structure of the hydroxybenzoic acid mitochondria-directed AntiOxBEN2 and (**B**) experimental design. To evaluate the effect of AntiOxBEN2 on bovine sperm capacitation and fertilization processes, this molecule was added to the capacitation (CAP) and/or to the fertilization (FERT) media, at the concentration of 0 (control), 1, and 10 µM, giving rise to seven groups. CAPcontrol: capacitation medium without supplementation; CAP1: capacitation medium supplemented with 1 μM of AntiOxBEN2; CAP10: capacitation medium supplemented with 10 μM of AntiOxBEN2; FERTcontrol: fertilization medium without supplementation; FERT1: fertilization medium supplemented with 1 μM of AntiOxBEN2; FERT10: fertilization medium supplemented with 10 μM of AntiOxBEN2.

The synthetic strategy and procedures used in the synthesis of the mitochondriotropic antioxidant AntiOxBEN2 (Figure 1A) have been previously described [4].

### 2.3. Experimental Design

To evaluate the effect of a novel mitochondrial-directed antioxidant molecule, AntiOxBEN2 [4], on both bovine sperm capacitation and fertilization processes and subsequent embryonic development, this molecule was added to the capacitation (CAP) and/or to the fertilization media, at the concentration of 0 (control), 1, and 10 µM (based on previous doses successfully applied during oocyte maturation [20]). Firstly, thawed bovine sperm from two bulls (pools of four ejaculates from the same bull) were submitted to the swim-up process in the CAP medium (CAPcontrol, CAP1, and CAP10, 6–8 replicates) for 1 h and then evaluated for motility (visual and computer-assisted sperm analysis (CASA)), vitality (eosin-nigrosine dye), mitochondrial membrane potential (JC1), and function (ATP and ROS production, basal metabolism—(Seahorse Xfe96). 

The same concentrations of AntiOxBEN2 were also supplemented to the fertilization medium (FERT) during the co-incubation of in vitro matured oocytes and capacitated spermatozoa (6 replicates, pool of four ejaculates per bull for each replicate), giving rise to 7 groups (Figure 1B) as follows: (1): CAPcontrol × FERTcontrol—FERT and CAP without supplementation; (2): CAPcontrol × FERT1—CAP without supplementation and FERT with 1 µM of AntiOxBEN2; (3): CAPcontrol × FERT10—CAP without supplementation and FERT with 10 µM AntiOxBEN2; (4): CAP1 × FERTcontrol—CAP with 1 µM of AntiOxBEN2 and FERT without supplementation; (5): CAP1 × FERT1—CAP and FERT with 1 µM of AntiOxBEN2; (6): CAP10 × FERTcontrol—CAP with 10 µM of AntiOxBEN2 and FERT without supplementation; and, finally, (7): CAP10 × FERT10—CAP and FERT with 10 µM of AntiOxBEN2.

Bovine oocytes (n = 1864) collected in 6 sessions were previously matured for 22 h and randomly distributed into the 7 groups prior to insemination with the capacitated spermatozoa. Afterwards, the embryonic development was evaluated.

### 2.4. Oocyte Collection and In Vitro Maturation

Bovine ovaries were harvested at the local abattoir, immediately after slaughter, and were placed in Dulbecco’s Phosphate Buffered Saline (PBS), at 38 °C, to be transported to the laboratory. PBS was previously supplemented with 0.15% bovine serum albumin (BSA, *w*/*v*) and 0.05 mgmL^−1^ kanamycin. Upon reaching the laboratory, the ovaries were washed, and the cumulus oocyte complexes (COC) of the follicles with a diameter between 2–6 mm were aspirated. Then, oocytes with uniformly granular cytoplasm and surrounded by at least 3 layers of compact cumulus cells were selected for maturation [21]. The COCs were matured in tissue culture medium 199 (TCM199) with 10% serum, 10 ng mL^−1^ epidermal growth factor (EGF), 0.2 mM sodium pyruvate, and antibiotics, in an incubator at 38.8 °C with an atmosphere saturated with humidity and 5% CO_2_, for 22 h [22].

### 2.5. Sperm Capacitation and Evaluation

In order for bovine spermatozoa to acquire the ability to penetrate and fertilize the oocyte, they have to undergo a maturation process called sperm capacitation [23]. For this, the swim-up method was used. Four frozen semen straws from each bull of proven fertility were thawed at 37 °C for 30 s. Then spermatozoa were submitted to the swim-up process in capacitation medium (modified Tyrode’s medium (TALP) without calcium supplemented with 2.4 mM of caffeine) [24], supplemented or not with the antioxidant molecule according to the experimental design. Briefly, thawed semen was carefully deposited, in equal doses (100 µL), on the bottom of the previously prepared capacitation medium-containing tubes (1.2 mL) and placed in the incubator for 1 h, in an atmosphere of 5% CO_2_, at 38.8 °C and with maximum humidity. During this period, the swim-up, a process by which the most active spermatozoa migrate to the surface of the liquid, concentrating there, occurred. Then, the upper layer was centrifuged at 225× *g* for 10 min and the supernatant was rejected. The remaining pellet of spermatozoa was evaluated and used to fertilize the oocytes in the fertilization medium. 

#### 2.5.1. Sperm Motility Assessment

Pre swim-up spermatozoa motility was visually analyzed. Post swim-up spermatozoa motility was analyzed visually and using CASA. The percentage of progressively motile spermatozoa was subjectively determined by the visual estimation of two experienced researchers (adapted from Pereira et al. [25]). Then, the ISAS-V1 CASA System (Proiser, Valencia, Spain) was used to assess sperm motility and kinematics. CASA analyses were performed recording five microscope fields with at least 200 cells per sample. In each sample, total and progressive motile (%), average path velocity (VAP, µms^−1^), straight-line velocity (VSL, µms^−1^), curvilinear velocity (VCL, µms^−1^), amplitude of lateral head displacement (ALH, µm), beat cross frequency (BCF, Hz), straightness, linearity, rapid, medium, slow, and static motile sperm were analyzed. 

#### 2.5.2. Sperm Vitality Assessment

Sperm viability and morphology were examined using the eosin-nigrosine dye [25]. Briefly, after the swim-up process, equal volumes of capacitated sperm samples and eosin-nigrosine dye were mixed on a microscope slide where it was smeared by sliding a cover slip in front of it. Then smears were air-dried and examined directly. At least 100 sperm cells from each sample were assessed at a magnification of 1000× under oil immersion. Spermatozoa that were white (unstained) were classified as live and those that showed any pink or red coloration were classified as dead. Moreover, sperm morphological abnormalities (tail, midpiece, and head) were evaluated.

#### 2.5.3. Mitochondrial Membrane Potential

The mitochondrial membrane potential (MMP) was determined using the florescent probe 5,5′,6,6′-tetrachloro-1,1′,3,3′-tetraethylbenzimidazolcarbocyanine iodide (JC-1, Invitrogen), as an indicator of mitochondrial activity. This probe shows fluorescence changes associated with the inner mitochondrial transmembrane potential, depicting orange/red fluorescence in fully functional midpiece mitochondria and green fluorescence in cells with lower inner mitochondrial membrane potential [26]. After subjecting the spermatozoa to the different treatments (CAPcontrol, CAP1 and CAP10) during the swim-up process, they were incubated with 5 μgmL^−1^ of JC-1 in Hanks solution supplemented with HEPES and BSA for 30 min at 38.8 °C and 5% CO_2_ in humidified air in the dark. Then, sperm samples (5.5 µL at 38.8 °C) were placed on glass microscope slides (Normax, ref. 1.5470217C) with coverslips, and observed in a fluorescence microscope (Olympus BX51) using the blue fluorescence filter (BP 470-490, objective UPlanFI 20×/0.50). Staining patterns were identified in 100 spermatozoa in each sample.

#### 2.5.4. Sperm Intracellular Oxidative Stress

Swimmed-up spermatozoa subjected to the different treatments (CAPcontrol, CAP1, and CAP10) were seeded in 96-well plates. Intracellular oxidative stress was assessed using the report molecule CM-H2DCFDA (5-(and-6)-chloromethyl-2′,7′-dichlorodihydrofluorescein diacetate, acetyl ester) (Life Technologies, Invitrogen). Briefly, cells were loaded with 5 μM of CM-H2DCFDA in assay buffer (NaCl 120 mM, KCl 3.5 mM, NaHCO3 5 mM, NaSO4 1.2 mM, KH2PO4 0.4 mM, and HEPES 20 mM supplemented with CaCl_2_ 1.3 mM, MgCl2 1.2 mM, and sodium pyruvate 10 mM, pH 7.4) at 38.5 °C and 5% CO_2_ in the dark for 15 min. Then, fluorescence signals were measured with 520 nm excitation and 620 nm emission wavelengths using a microplate reader (Cytation 3; BioTek Instruments, Winooski, VT, USA). Results were normalized for cell mass content at the end, using the sulforhodamine B (SRB) assay.

#### 2.5.5. Cell Mass

Swimmed-up spermatozoa subjected to the different treatments were used for cell mass determination based on the measurement of the cellular protein content with the SRB assay [27]. Cells were fixed by adding 50 µL of 60% trichloroacetic acid (TCA) and were stored overnight at 4 °C. The fixation solution was then discarded, and the plates were dried at 37 °C. Then, 150 µL of 0.05% SRB in 1% acetic acid solution was added and incubated at 37 °C for 1 h. The wells were washed with 1% acetic acid in water and dried. Then, 100 μL of Tris (pH 10) was added and the plates were stirred for 15 min and optical density was measured at 540 nm in the Biotek Cytation 3 reader (Biotek Instruments, Winooski, VT, USA).

#### 2.5.6. Intracellular ATP Levels

Swimmed-up spermatozoa from different treatments were seeded in 50 µL of culture medium, in a white opaque-bottom, 96-well plate. Intracellular ATP levels were measured using the CellTiter-Glo Luminescent Cell Viability Assay (Promega, WI, USA) following the manufacturer’s instructions. Briefly, 50 µL of medium containing CellTiter-Glo Reagent (CellTiter-Glo Buffer + CellTiter-Glo Substrate) was added to the cells. Contents were mixed for 2 min on an orbital shaker to induce cell lysis and, after 10 min of incubation at 22 °C, the luminescence signal was monitored in a Cytation 3 reader (Biotek Instruments, Winooski, VT, USA). An ATP standard curve was also generated following the manufacturer’s instructions.

#### 2.5.7. Cellular Oxygen Consumption Rate

After capacitation, spermatozoa (control, CAP1, and CAP10) were centrifuged and placed in a 96-well plate (pre-coated with collagen I at 0.15 mg mL^−1^) in synthetic oviductal fluid (SOF) medium at a density of 1 × 10^6^ cells/100µL/well (16 wells for each group and bull). Cellular oxygen consumption was measured at 37 °C using a Seahorse XFe96 Extracellular Flux Analyzer (Agilent, Germany). 

The day before, an XFe96 sensor cartridge for each cell plate was placed in a 96-well calibration plate containing 200 µL/well calibration buffer and it was left to hydrate at 37 °C. For the oxygen consumption rate (OCR) measurements, 1 µM rotenone and 1 µM antimycin A were injected into reagent delivery port A. Then, 25 µL of compounds was pre-loaded into the ports of each well in the XFe96 sensor cartridge. The sensor cartridge and the calibration plate were loaded into the XFe96 Extracellular Flux Analyzer for calibration. The calibration plate was replaced with the study plate when the calibration was complete. Three baseline rate measurements of OCR were made using a 3 min mix, 5 min measure cycle. The compounds were then pneumatically injected by the XFe96 Analyzer into each well, were mixed, and OCR measurements were taken using a 3 min mix, 5 min measure cycle. Results were analyzed by using the Software Version Wave Desktop 2.6. [28].

### 2.6. Oocyte In Vitro Fertilization and Embryo Development

Swimmed-up spermatozoa (1–2 µL to obtain a final concentration of 2 × 10^6^ spzmL^−1^) were added to droplets of the fertilization medium (40 µL), containing 10 oocytes each. The in vitro fertilization medium consisted of modified Tyrode’s medium supplemented with 5.4 USP mL^−1^ of heparin, 10 mM of penicillamine, 20 mM of hypotaurine, and 0.25 mM of epinephrine [22], and it was also supplemented or not with AntiOxBEN2 according to the experimental design. The spermatozoa and oocytes were co-incubated for 20 h. Then, denuded presumptive zygotes were placed in 25 µL droplets of SOF supplemented with the MEM-non-essential Amino Acid Solution, BME-Amino acids solution, glutamine, glutathione, and BSA and were cultured at 38.8 °C in a humidified atmosphere with 5% O_2_, 5% CO_2_, and 90% N_2_. [22] After assessing cleavage at 48 h post insemination, embryo development proceeded in SOF BSA plus 10% fetal calf serum (FCS) (IVF = day 0) for ten days in the incubator at the same conditions.

Embryo development was evaluated by the cleavage, day 7, and hatched rates and quality of produced embryos. Cleavage (2–4 cell embryos) rates were calculated as a proportion of inseminated oocytes. D7/8 embryo (morula and blastocysts, with most embryos being at the blastocyst stage) developmental rates were calculated as a proportion of cleaved embryos, while the rates of hatched embryos were calculated as a proportion of embryos of day 7/8 that effectively hatched from the zona pellucida until D12 [29]. Moreover, D7 embryos were classified as good, fair, and bad according to International Embryo Technology Society guidelines [30]. The rate of embryonic development was also assessed on D7 and 9, identifying morulae (M), young blastocysts (YBL), blastocysts (BL), expanded blastocysts (EBL), and hatched blastocysts (HBL) [29].

### 2.7. Statistical Analysis

The procedure MIXED of the Statistical Analysis Systems Institute (SAS Inst., Inc., Cary, NC, USA) was used to analyze sperm parameters. The mixed linear model included the treatment (CAP) and bull as fixed effects. In addition, the means for each treatment were calculated and compared using the PDIFF test.

Embryo production data were analyzed with the procedure GLIMMIX using the binary distribution and the logit as the link function. The generalized linear mixed model included treatment (CAPxFERT) and session as the fixed and random effects, respectively. Furthermore, comparisons between groups were performed using the PDIFF test. Data were analyzed in GraphPad Prism 8.0.2 software (GraphPad Software, Inc., San Diego, CA, USA), with all results being expressed as means ± standard error of the mean (SEM) for the number of experiments indicated. Values were considered statistically different when *p* ≤ 0.05.

## 3. Results

### 3.1. AntiOxBEN2 Dose-Dependently Decreased Sperm Motility

After thawing, the bull semen was immediately evaluated for individual motility (bull 1 = 76.5 ± 2.19% and bull 2 = 72.5 ± 3.26%) and then submitted to the swim-up process in the capacitation medium, supplemented or not (control, CAPcontrol) with the AntiOxBEN2 (1 and 10 μM, CAP1 and CAP10, respectively). The sperm motility and kinematic parameters are represented in Table 1.

The presence of AntiOXBEN2 during capacitation influenced the motility of spermatozoa (*p* ≤ 0.03) evaluated either by using CASA or visually. In fact, AntiOxBEN2 induced a dose-dependent decrease in progressive motility of spermatozoa (CAP control vs. CAP10, *p* = 0.009, Table 1). Similarly, AntiOxBEN2 also dose-dependently affected (*p* = 0.004) visual spermatozoa individual motility, with the highest dose impairing this motility compared to the 1 μM (CAP1, *p* = 0.04) and control (*p* = 0.001) groups (Table 1).

A bull effect was observed in the number of spermatozoa classified with medium velocity (*p* = 0.02). All other measured parameters (Table 1) showed no significant effects, neither for the bull nor for the treatment with the molecule under study.

### 3.2. AntiOxBEN2 Did Not Alter Semen Vitality nor Induced Sperm Abnormalities

Sperm vitality and morphology directly impact sperm quality [31]. To determine whether AntiOxBEN2 induced structural abnormalities in spermatozoa during capacitation, the sperm normality and head, midpiece, or tail defect rates were quantified (*p* > 0.05, Table 2). Interestingly, AntiOxBEN2 did not induce spermatozoa structural abnormalities. Moreover, a bull effect was not observed for any of the evaluated parameters (*p* > 0.05).

### 3.3. AntiOxBEN2 Improved Sperm Mitochondrial Membrane Potential

**Figure 2:**
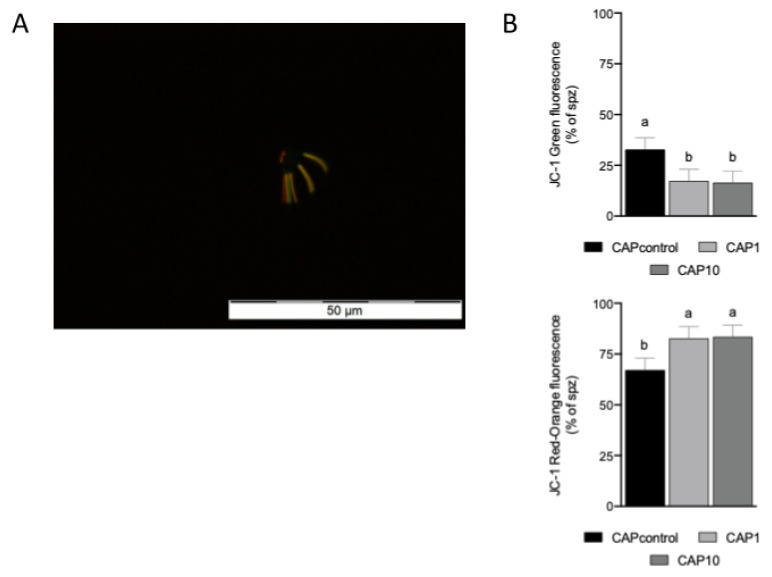
Effects of the mitochondria-targeted hydroxybenzoic acid derivative AntiOxBEN2 supplementation on mitochondrial membrane potential (MMP) of spermatozoa during the capacitation process. (**A**) Typical image of bovine spermatozoa stained with the florescent probe 5,5′,6,6′-tetrachloro-1,1′,3,3′-tetraethylbenzimidazolcarbocyanine iodide (JC-1, Invitrogen), as an indicator of mitochondrial activity. Labeled sperm showed a clear midpiece-only staining pattern; the midpiece was either homogeneously green (low DΨmit) or with speckles of yellow/orange/red (high DΨmit). (**B**) Percentage of spermatozoa emitting yellow/orange/red (high DΨmit) (down panel) and green (low DΨmit) (upper panel) fluorescence after capacitation in the presence or absence of AntiOxBEN2 (1 and 10 µM) supplementation. The data (mean ± SEM, 6 replicates) obtained with AntiOxBEN2 were compared to control using the mixed procedure/PDIFF of SAS. Significant differences between the indicated conditions are marked by different letters (a ≠ b, *p* < 0.05). CAPcontrol: capacitation medium without supplementation; CAP1: capacitation medium supplemented with 1 μM of AntiOxBEN2; CAP10: capacitation medium supplemented with 10 μM of AntiOxBEN2.

Sperm motility depends on mitochondrial function and the measurement of mitochondrial membrane potential (MMP) better accounts for the function of this intracellular organelle [12,26]. Consequently, the impact of AntiOxBEN2 on MMP of spermatozoa during capacitation was measured using the JC-1 fluorescent dye. Interestingly, a significant effect (*p* = 0.003, Figure 2A) of AntiOxBEN2 on mitochondrial membrane potential was identified. AntiOxBEN2 (1 μM (CAP1, *p* = 0.003), and 10 μM (CAP10, *p* = 0.0021)) improved the number of spermatozoa emitting a bright red-orange fluorescence when compared to the control group (Figure 2B), suggesting an increase in sperm mitochondrial membrane potential. Moreover, a bull effect was not observed (*p* > 0.05).

### 3.4. AntiOxBEN2 Dose-Dependently Increased Sperm Cellular (Basal) Oxygen Consumption

**Figure 3:**
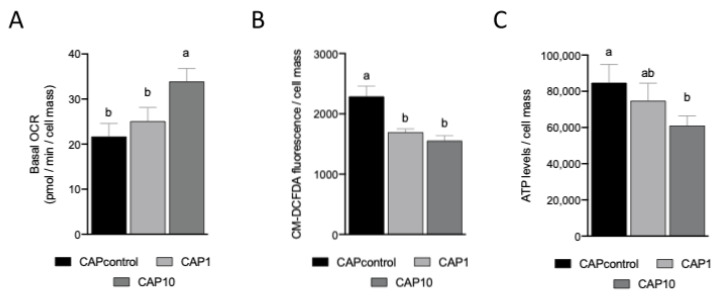
Effects of the mitochondria-targeted hydroxybenzoic acid derivative AntiOxBEN2 supplementation on mitochondrial oxygen consumption, ATP, and ROS levels of spermatozoa during the capacitation process. (**A**) The basal oxygen consumption rate (OCR) of bovine spermatozoa in the presence or absence of AntiOxBEN2 (1 and 10 µM) supplementation during the capacitation process was analyzed by using the Seahorse XFe96 Extracellular Flux Analyzer. Data are mean ± SEM of 16 replicates for each bull, and the results are expressed as pmol O2/min/cell mass. (**B**) The average fluorescence signal of the cellular CM-H2DCFDA oxidation product of bovine spermatozoa in the presence or absence of AntiOxBEN2 (1 and 10 µM) supplementation during the capacitation process. Data are mean ± SEM of 8 replicates, and the results are expressed as CM-H2DCFDA fluorescence per spermatozoa cell mass. (**C**) Intracellular ATP levels of bovine spermatozoa in the presence or absence of AntiOxBEN2 (1 and 10 µM) supplementation during the capacitation process. Data are mean ± SEM of 8 replicates, and the results are expressed as ATP levels per spermatozoa cell mass. Significant differences between the indicated conditions are marked by different letters (a ≠ b, *p* < 0.05); CAPcontrol: capacitation medium without supplementation; CAP1: capacitation medium supplemented with 1 μM of AntiOxBEN2; CAP10: capacitation medium supplemented with 10 μM of AntiOxBEN2.

Mitochondrial oxygen consumption is a sensitive indicator of spermatozoa health since it is positively correlated with traditional measures of sperm function, including motility and viability [32]. Consequently, the impact of AntiOxBEN2 on mitochondrial oxygen consumption of spermatozoa during capacitation was measured through variations on cellular oxygen consumption rate (OCR) using the Seahorse XF-96 Extracellular Flux Analyzer. Interestingly, AntiOxBEN2 dose-dependently increased spermatozoa basal OCR (control = 21.7 ± 2.88 pmol/min; CAP1 = 25.1 ± 3.05 pmol/min; and CAP10 = 33.9 ± 2.88 pmol/min, *p* = 0.004 and *p* = 0.04, respectively) (Figure 3A). Similar to MMP measurements, a bull effect was not observed for cellular OCR measurements (*p* > 0.05). 

### 3.5. AntiOxBEN2 Decreased Sperm Cellular Oxidative Stress

Sperm capacitation can be increased by ROS and decreased by antioxidants. However, the threshold for ROS levels that may induce functional sperm ability or may lead to dysfunctional sperm is still an unsolved question [1,33]. We have measured the impact of AntiOxBEN2 on spermatozoa oxidative stress after capacitation using a fluorescent reporter molecule (CM-H2DCFDA). Remarkably, AntiOxBEN2 decreased spermatozoa intracellular oxidative stress (CAP1 = 1696.4 ± 90.75 fluorescence/cell mass, *p* < 0.0001; and CAP10 = 15,554.7 ± 90.75 fluorescence/cell mass, *p* < 0.0001), when compared to control (2290.1 ± 90.75 fluorescence/cell mass, Figure 3B). Moreover, a bull effect was observed in the measurements (*p* = 0.006), as the bull 1 presented significantly higher intracellular oxidative stress (2046.8 ± 87.58), when compared to bull 2 (1647.3 ± 87.58 fluorescence/cell mass).

### 3.6. AntiOxBEN2 Dose-Dependently Decreased Sperm Intracellular ATP Levels

The sperm midpiece contains mitochondria that produce chemical energy in the form of ATP via oxidative phosphorylation (OXPHOS). ATP may also be produced by glycolysis. Although the relative contribution of energy for motility from these two pathways differs between species, proper functioning of the mitochondria appears to be consistently vital for normal sperm motility [34,35]. Therefore, we tested the effects of AntiOxBEN2 on intracellular ATP levels of spermatozoa after capacitation. AntiOxBEN2 dose-dependently decreased spermatozoa intracellular ATP levels (Figure 3C), when compared to control (84,762.0 ± 8574.93 ATP levels/cell mass); however, this effect was only significant when CAP was supplemented with 10 μM of AntiOxBEN2 (CAP10 = 61,110.0 ± 8574.93 ATP levels/cell mass, *p* = 0.008). Similarly, a bull effect was also observed in ATP levels (*p* = 0.003). In fact, the bull 1 presented higher intracellular ATP levels (83,705.0 ± 7939.39), when compared to bull 2 (63,397 ± 7939.39 ATP levels/cell mass) (*p* = 0.003).

### 3.7. AntiOxBEN2 Dose-Dependently Increased the Number of Produced Embryos

**Figure 4:**
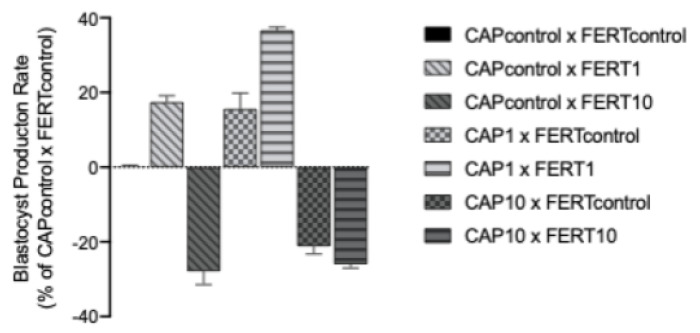
Effects of the mitochondria-targeted hydroxybenzoic acid derivative AntiOxBEN2 supplementation during capacitation and fertilization processes on Day 7 embryo production rates. Results from all groups were normalized by using the CAPcontrol × FERTcontrol group blastocyst rate. CAPcontrol: capacitation medium without supplementation; CAP1: capacitation medium supplemented with 1 μM of AntiOxBEN2; CAP10: capacitation medium supplemented with 10 μM of AntiOxBEN2; FERTcontrol: fertilization medium without supplementation; FERT1: fertilization medium supplemented with 1 μM of AntiOxBEN2; FERT10: fertilization medium supplemented with 10 μM of AntiOxBEN2.

Various studies have shown that adding antioxidants into embryo culture medium improves in vitro embryo development [36]. Here, we evaluated the effects of AntiOxBEN2 on embryonic development and quality after supplementation in capacitation medium (CAP) of spermatozoa, fertilization medium (FERT), or both. The supplementation of both the capacitation and fertilization media with AntiOxBEN2 influenced the cleavage rate (*p* = 0.02, Table 3), but had no significant effect in the embryo production rate at D7 (*p* > 0.05), nor in the hatched embryo rate (*p* > 0.05). Nevertheless, the supplementation of both media with 1 µM of AntiOxBEN2 more than doubled the number embryos produced at D7 compared to the 10 µM dose. Moreover, the supplementation of CAP and/or FERT media with 1 μM of AntiOxBEN2 always improved blastocyst production rate compared to control (Figure 4). On the contrary, the highest dosage decreased the blastocyst production rate.

The CAP1 × FERT1 group showed a higher cleavage rate when compared to the CAPcontrol × FERTcontrol (*p* = 0.01), CAP10 × FERTcontrol (*p* = 0.046), CAP1 × FERTcontrol (*p* = 0.01), and CAPcontrol × FERT10 (*p* = 0.001) groups. Moreover, CAPcontrol × FERT1 and CAP10 × FERT10 also had higher (*p* ≤ 0.04) cleavages rates when compared to CAPcontrol × FERT10 (Table 3).

Supplementation of the fertilization and/or capacitation media showed no significant influence (*p* > 0.05) on the quality of the embryos produced, i.e., the rate of embryos produced in grade 1 (Good), grade 2 (Fair), or grade 3 (Bad). No differences (*p* > 0.05) were observed in the different embryonic stages (morula, blastocysts, or hatched) of produced embryos either at day 7 or day 9.

## 4. Discussion

Our results showed for the first time that the mitochondrial-targeted antioxidant, AntiOxBEN2, influenced both bovine sperm capacitation and fertilization processes, thereby improving early embryonic development. Moreover, the positive effect of this antioxidant in the prevention of oxidative stress in bovine gametes was also confirmed. Indeed, the supplementation of the capacitation and fertilization media with AntiOxBEN2 induced a substantial reduction in ROS production by spermatozoa reverberating on the embryos’ cleavage rate. More significant results were obtained when both media were supplemented with 1 μM AntiOxBEN2 compared to the untreated groups, except for the CAPcontrol × FERT1 group, where the FERT medium was supplemented and that CAP was not also presenting high cleavage rates. The data clearly suggest that the antioxidant AntiOxBEN2 exerts its effects on spermatozoa and oocytes, as previously described by Teixeira et al. [20]. However, in this case, the antioxidant molecule was only added to the maturation medium of oocytes, and not to the fertilization medium, as in the present study. A beneficial effect of AntiOxBEN2 linked with higher cleavage rates was identified. Other substances, such as glutathione [37] and L-arginine [38], were previously tested in the fertilization medium, and while the latter showed beneficial effects on the cleavage and blastocyst production rates, glutathione had no effect, at least at the concentrations tested. Nowadays, several reports concerning the supplementation of extenders, IVF, and cryopreservation media for gametes and embryos with antioxidants have been published [37,38,39,40]. While some have identified beneficial effects, others could not find such effects. Recently, the mitochondria-targeted antioxidant MitoQ10 was used to prevent oxidative stress during oocyte maturation and has shown positive results, increasing the rate of produced blastocysts [41].

Dietary polyphenol antioxidants and derivatives, such as AntiOxBEN2, can prevent and minimize oxidative stress events acting primarily as ROS scavengers but also minimizing metal-dependent (Cu+ and Fe2+) hydroxyl radical formation, mainly through a chelation mechanism, and modulating ROS-removing enzymes and/or inhibition of ROS producing enzymes [4,42]. The ability of a phenolic antioxidant to perform its function, i.e., its capability to transfer a hydrogen atom or an electron to a stable free radical, is a result of the deactivation of this radical by the antioxidant. Therefore, in theory, molecules with higher antioxidant power show a higher percentage of radical inhibition [4]. When compared to MitoQ10, AntiOxBEN2 presented a higher ability to chelate ferrous iron, a capacity that was superior to that observed for its precursor gallic acid [4]. AntiOxBEN2 also has the capacity to prevent mitochondrial lipid peroxidation, exhibiting lower toxicity profiles than MitoQ in different cell types [4], highlighting the considerable potential of mitochondria-targeted phenolic antioxidants to improve ART outcomes.

The capacity of AntiOxBENs to be accumulated by energized mitochondria was confirmed in isolated rat liver mitochondria [4]. A positive effect of both doses of AntiOxBEN2 on the mitochondria of bovine spermatozoa was identified in the present study. This antioxidant increased the number of spermatozoa emitting a bright red-orange fluorescence after JC1 staining compared to control, without interfering with their vitality. Previous studies have identified JC1 dye as a powerful tool to differentiate the quality of the mitochondrial function in bovine and human sperm related to fertility rates [43,44,45]. Accordingly, our goal was to test the performance of the antioxidant AntiOxBEN2 specially developed to act in mitochondria [4,15], the main production site for ROS in bovine spermatozoa [9,34], in two crucial phases of in vitro artificial insemination processes: in sperm capacitation and in the moment of fertilization. Semen capacitation and acrosomal exocytosis are two crucial processes for semen quality and, consequently, for its capacity for fertilization, and are therefore closely related to male fertility [38,46]. It is also widely known that embryonic development is related to the quality of gametes [47]. In in vitro methods, male and female gametes are exposed to high levels of ROS. These ROS levels reduce sperm motility, induce lipid peroxidation, and generate DNA damage leading to sperm death. Moreover, they decrease the sperm-oocyte fusion capacity and delay embryonic development [37]. In the present study, both AntiOxBEN2 doses (1 and 10 µM) reduced ROS production by spermatozoa demonstrating that it was possible to target mitochondrial oxidative stress in the male gametes. By specifically reducing mitochondrial ROS production in gametes, AntiOxBEN2 played an important role in maintaining sperm function [1,4,15]. These encouraging results may contribute to improving ART success and to the prevention/treatment of infertility problems. 

Mitochondria are the site for critical metabolic and physiological processes such as the oxidative phosphorylation (OXPHOS), which couples substrate oxidation to ATP synthesis. During OXPHOS, electrons are transferred from NADH or succinate to electron carriers and oxygen. Such electron transfer is mediated by oxido-reductive reactions of the electron transport in the inner mitochondrial membrane and cristae. The energy harvested during these oxido-reductive reactions is stored in a proton gradient across the inner mitochondrial membrane that is dissipated during ATP production [15,48]. In this study, the function of OXPHOS was investigated in bovine spermatozoa by monitoring oxygen consumption and ATP production. Basal respiration of spermatozoa tends to increase dose-dependently with the supplementation of AntiOxBEN2. However, the production of ATP was the lowest at the highest dose of AntiOxBEN2. Moreover, we also observed that at the highest AntiOxBEN2 dosage, the motility of spermatozoa was reduced, a process probably related with the observed decrease of ATP production. Depending on species and environmental conditions, sperm cells can obtain their energy through two main pathways, OXPHOS and glycolysis. In bovine spermatozoa, OXPHOS is predominant, and the majority of the sperm’s energy is directed towards motility. Additionally, swim-up selected sperm, as used in the present study, show higher ATP production via OXPHOS, but also higher ATP expenditure [34,35]. Therefore, the metabolic activity of sperm is considered an essential indicator for sperm quality that should be reflected on their potential for fertilization and embryonic development. In the present study, differences between bulls were identified for oxidative stress and ATP production, but not for OCR, requiring further studies.

Comparing the two groups in which both media were supplemented, with 1 and 10 μM of AntiOxBEN2, the number of embryos produced at D7 at the concentration of 1 μM was more than double than those observed at 10 μM. Therefore, although the cleavage rates of these two groups were similar, the concentration of 10 μM seemed already harmful in later phases. This detrimental effect at 10 μM was also identified through the reduction of spermatozoa motility and ATP production after the capacitation process. Both oocytes and spermatozoa are very sensitive to cytotoxicity, which is negatively reflected in their developmental potential [10,49]. Although the doses tested herein were not toxic to rat embryonic cardiomyoblasts, human neonatal dermal fibroblasts, and hepatocellular carcinoma cells [4], our results suggest using the lowest dosage when dealing with germplasm. The extent of the uptake of a mitochondriotropic antioxidant that use TPP+ as carriers depends on the plasma ΔΨ and ΔΨm, the volume of the cell, the external media, and the number of mitochondria within a given cell. Consequently, the concentration of the compound found within the mitochondria can substantially differ according to cell types [50]. Moreover, according to Teixeira et al. [4], higher concentrations of AntiOxBEN2, above those necessary to exert the antioxidant effect, presented a toxicity effect. Further dose-responses studies may be performed to establish the optimal dose to potentiate the AntiOxBEN2 effect on different in vitro embryo production steps.

By analyzing our results, it was possible to observe that AntiOxBEN2, when supplemented to the fertilization medium, was more effective than when added only to the sperm capacitation medium. This behavior may be ascribed to the locale of action, as when added during the fertilization process, it acts on both gametes and, in this case, it continues to act on both for twenty hours, until they are washed and placed in the SOF droplets. The length of permanence of the AntiOxBEN2 on oocyte and zygote mitochondria is unknown and should also be further investigated. The supplementation of the embryo culture media with this antioxidant could probably also be beneficial, as shown in the present work and by Teixeira and collaborators [20].

In the present study, when the antioxidant was only added to the CAP medium, a positive effect on the fertilization process was not clearly identified, which is a problem that can be related to the incubation time of semen with AntiOxBEN2, which may have been short, and/or the high quality of the semen used. Other authors [39] showed promising results when longer incubation times of 120 and 240 min of sperm with antioxidants were performed. Moreover, other studies showed that men with semen of low quality (infertile) have higher levels of ROS compared to men with semen of high quality (fertile) [6]. Equally, the bull sperm quality declines with advancing age, which is strongly associated with increased oxidative damage of the spermatozoa plasma membrane and DNA [5]. Infertility seems to be associated with high ROS and oxidative stress levels, leading to more significant cellular damage and decreased sperm viability. Thus, we can speculate that the effect of AntiOxBEN2 would be more noticeable in semen with less viability since the ROS levels would expectedly be higher. This higher level of ROS is due, as previously described, to an imbalance between their production and the action of endogenous antioxidants [51], a situation where the addition of exogenous antioxidants can be beneficial. In a future study, we should also test the effect of the antioxidant with semen of inferior quality than the one used in the present study. Longer incubation times for spermatozoa and its introduction in different in vitro embryo production stages may be attempted. Another hypothesis would be to add AntiOxBEN2 before semen cryopreservation to the prevent damage caused by the ROS produced in the freeze-thaw processes. In addition, other molecular studies should be implemented to deepen the knowledge about the mechanism of action of AntiOxBEN2 on embryo development.

## 5. Conclusions

Due to the rapid development of assisted reproduction techniques and emerging infertility problems worldwide, it has become essential to develop effective antioxidants capable of effectively combating in vivo and in vitro ROS production and consequent reproductive function impairment. Our data establish that AntiOxBEN2, a novel mitochondrial-directed antioxidant, has beneficial effects on reducing ROS levels and on the mitochondrial membrane potential increment, and, consequently on sperm quality, when added to the capacitation medium. Moreover, it was also possible to observe that AntiOxBEN2 has a positive effect on cleavage rates and the number of D7 produced embryos, when supplemented to the fertilization medium. These promising results demonstrate a beneficial effect of AntiOxBEN2 at 1 µM dosage on both sperm capacitation and fertilization processes, thus improving embryonic development. Further studies should be conducted to better understand the benefits of AntiOxBEN2 supplementation on sperm quality and metabolism.

## Figures and Tables

**Table 1:** Evaluation of the effect of AntiOxBEN2 on the kinematic parameters of semen using Computer Assisted Sperm Analysis (CASA) software and visual observation (6 sessions).

Kinematic Parameters	Groups		
	CAPcontrol	CAP1	CAP10
Total Motility (%)	79.6 ± 7.03	75.8 ± 7.03	69.8 ± 7.03
Progressive Motility (%)	42.8 ± 5.85 ^a^	37.9 ± 5.85 ^ab^	28.5 ± 5.85 ^b^
Visual Motility (%)	56.5 ± 5.16 ^a^	50.7 ± 5.16 ^a^	44.0 ± 5.16 ^b^
VCL (µms^−1^)	114.6 ± 14.4	99.5 ± 14.4	97.5 ± 14.4
VAP (µms^−1^)	59.1 ± 7.02	51.6 ± 7.02	50.5 ± 7.02
Slow motile spz (n)	4.9 ± 2.47	8.3 ± 2.47	11.8 ± 2.47
Medium motile spz (n)	20.9 ± 6.05	26.4 ± 6.05	25.1 ± 6.05
Rapid motile spz (n)	145.1 ± 35.16	136.4 ± 35.16	92.1 ± 35.16
VSL (µms^−1^)	45.2 ± 6.21	37.4 ± 6.21	35.6 ± 6.21
ALH (µm)	2.8 ± 0.30	2.5 ± 0.30	2.6 ± 0.30
Linearity	39.1 ± 1.95	37.0 ± 1.95	37.3 ± 1.95
Straightness	75.4 ± 2.72	71.6 ± 2.72	70.8 ± 2.72
Wobble VAP/VCL	51.8 ± 1.23	51.5 ± 1.23	52.5 ± 1.23
BCF (Hz)	13.5 ± 1.18	13.3 ± 1.18	11.9 ± 1.18

Data are represented as mean ± standard error of mean. Different letters indicate significant differences (a ≠ b, *p* ≤ 0.05). VCL: curvilinear velocity; VAP: average path velocity; spz: spermatozoa; VSL: straight-line velocity; ALH: amplitude of lateral head displacement; BCF: beat cross frequency. CAPcontrol: capacitation medium without supplementation; CAP1: capacitation medium supplemented with 1 μM of AntiOxBEN2; CAP10: capacitation medium supplemented with 10 μM of AntiOxBEN2.

**Table 2:** Sperm vitality, head, intermediate piece and tail anomalies, concentration, and mitochondrial membrane potential after the capacitation with AntiOxBEN2.

Kinematic Parameters	Groups		
	CAPcontrol	CAP1	CAP10
Vitality (%)	47.3 ± 6.96	44.9 ± 6.96	45.8 ± 6.96
Normal spz (%)	79.8 ± 1.65	81.2 ± 1.65	80.5 ± 1.65
Head defect (%)	7.3 ± 0.84	7.4 ± 0.84	9.1 ± 0.84
Int.piece defect (%)	6.8 ± 1.17	5.8 ± 1.17	5.1 ± 1.17
Tail defect (%)	6.7 ± 0.88	6.2 ± 0.88	5.8 ± 0.88

Data are represented as mean ± standard error of mean. CAPcontrol: capacitation medium without supplementation; CAP1: capacitation medium supplemented with 1 μM of AntiOxBEN2; CAP10: capacitation medium supplemented with 10 μM of AntiOxBEN2.

**Table 3:** Effect of oocytes and semen treatment with different concentrations of AntiOxBEN2 on cleavage and embryo production rates (mean ± SEM, 6 sessions).

Groups	Inseminated Oocytes (n)	Cleavage (%)	D7 Embryos (n)	D7 Embryos (%)
CAPcontrol × FERTcontrol	306	61.8 ± 2.84 ^bc^	27	15.9 ± 2.85
CAPcontrol × FERT1	266	69.4 ± 2.86 ^ab^	29	18.4 ± 3.12
CAPcontrol × FERT10	243	57.2 ± 3.22 ^c^	13	10.9 ± 2.87
CAP1 × FERTcontrol	248	61.4 ± 3.14 ^bc^	25	17.7 ± 3.24
CAP1 × FERT1	264	72.7 ± 2.77 ^a^	37	21.6 ± 3.16
CAP10 × FERTcontrol	293	64.4 ± 2.85 ^bc^	20	12.2 ± 2.58
CAP10 × FERT10	244	66.9 ± 3.06 ^ab^	16	11.6 ± 2.66

Different letters indicate significant differences (a ≠ b ≠ c, *p* ≤ 0.05); CAPcontrol: capacitation medium without supplementation; CAP1: capacitation medium supplemented with 1 μM of AntiOxBEN2; CAP10: capacitation medium supplemented with 10 μM of AntiOxBEN2; FERTcontrol: fertilization medium without supplementation; FERT1: fertilization medium supplemented with 1 μM of AntiOxBEN2; FERT10: fertilization medium supplemented with 10 μM of AntiOxBEN2, D7: day seven.

## Data Availability

Data is contained within the article.
